# Hepatoprotective potential of *Fagonia olivieri DC*. against acetaminophen induced toxicity in rat

**DOI:** 10.1186/s12906-016-1445-x

**Published:** 2016-11-09

**Authors:** Umbreen Rashid, Muhammad Rashid Khan, Moniba Sajid

**Affiliations:** 1 Department of Environmental Sciences, GC Women University Sialkot, Sialkot, Pakistan; 2 Department of Biochemistry, Faculty of Biological Sciences, Quaid-i-Azam University, Islamabad, 45320 Pakistan

**Keywords:** Antioxidant, *Fagonia olivieri*, Hepatotoxicity, Total phenolic content

## Abstract

**Background:**

*Fagonia olivieri* (DC) being used for the treatment of diabetes, cancer, fever and claimed to be effective in many other stress related disorders. In this study we have evaluated the *F. olivieri* whole methanol extract and its derived fractions for various in vitro and in vivo antioxidant studies.

**Methods:**

The crude methanol extract of the whole plant of *F. olivieri* (FOM) and its derived fractions; n-hexane (FOH), chloroform (FOC), ethyl acetate (FOE), n-butanol (FOB) and aqueous (FOA) were evaluated for the total phenolic and flavonoid content and in vitro antioxidant abilities. The antioxidant effect of FOM was determined by acetaminophen-induced hepatotoxicity in Sprague–Dawley (*Rattus novergicus*) male rats. The methanol/fractions were also analysed by HPLC analysis for the presence of polyphenolics.

**Results:**

The total phenolic content of the samples ranged from 19.3 ± 0.529 to 106.2 ± 0.892 mg GAE/g extract while total flavonoid content 16.2 ± 0.881 to 50.1 ± 1.764 mg RTE/g extract, respectively. FOA showed highest radical scavenging activity for DPPH (IC_50_ = 55.2 ± 1.212 μg/ml), ABTS (IC_50_ = 90.2 ± 1.232 μg/ml) superoxide (IC_50_ = 37.1 ± 0.643 μg/ml) and for H_2_O_2_ (IC_50_ = 64 ± 1.463 μg/ml). FOE exhibited the highest antioxidant activities for phosphomolybdenum (IC_50_ = 78.2 ± 0.883 μg/ml) and for hydroxyl radical scavenging (IC_50_ = 82 ± 2.603 μg/ml). HPLC analysis of FOM and its derived fractions showed the presence of rutin, catechin and gallic acid. Elevated levels of AST, ALT, ALP, LDH and lipid profile in serum and lipid peroxidation and DNA damages in liver; while decreased activity level of CAT, SOD, GSH-Px, GR and reduced glutathione (GSH) concentration induced with acetaminophen in rat were reverted towards the control group with co-administration of FOM.

**Conclusion:**

Our results showed that *F. olivieri* is a potential source of natural antioxidants, which justifies its use in folklore medicine.

## Background

Free radicals are produced in living systems through normal metabolic processes of the body. The reactive oxygen species (ROS) mainly include hydroxyl radical (^•^OH), superoxide anion (O_2_^−^) and hydrogen peroxide (H_2_O_2_) are produced in small amount for different physiological functions [1, 2]. However, increased concentrations of free radicals may result into many disorders such as atherosclerosis, arthritis, Alzheimer disease, cancer etc. Radiations, pollutants, toxins, deep fried and spicy foods, chemicals and physical stress are the main sources of free radical generation. Free radicals can induce abnormal proteins, depletion of antioxidants, immune system and changes in gene expression [3]. The antioxidants such as polyphenolics can protect the biological systems against the harmful effects of oxidative processes [4]. Thus, increased consumption of plant derived products which are good sources of antioxidants is a convenient way to control the diseases induced with oxidative stress [5].

Antioxidants can be of synthetic origin and can be a great number of secondary metabolites isolated from plants, such as various phenolic compounds [5]. Synthetic antioxidants like butylated hydroxyanisole (BHA) and butylated hydroxytoluene (BHT) were found to have genotoxic effect [6]. Therefore, it is of considerable interest to explore the plant based derivatives for their antioxidant and other biological activities. During recent decades much interest in antioxidants especially in herbal medicines emanates from their long use in folklore medicine system and their prophylactic properties. Most of the population in developing countries depends upon medicinal plants for basic pharmaceutical care. The antioxidants possess multifacetedeness in their action and provide enormous magnitude in maintaining balance. Antioxidant constituents of herbal products may contribute towards the remedy from various ailments. Use of antioxidant rich diets in daily life has shown a negative association with morbidity and mortality [7].

Liver diseases remains a worldwide health problem and are mostly associated with oxidative stress and tissue injury. The use of synthetic medicine in treating liver diseases can at times have serious side effects. Acetaminophen is a substitute of aspirin and is used worldwide for its analgesic and antipyretic properties [8]. The chronic use of acetaminophen or intake in large doses is usually related with hepato- and nephrotoxicity in animals and humans [9]. Acetaminophen is metabolized in liver via cytochrome P450 pathway into a highly toxic metabolite, N–acetyl–p–benzoquinamine (NAPQI), which is usually conjugated with glutathione and excreted in urine [9]. Acetaminophen overdose can lead to mitochondrial dysfunction by draining stores of glutathione resulting in acute hepatic necrosis development [10]. Herbal treatments for many diseases such as hepatopathy are increasing in many countries. Most of the important drugs of the modern system of medicine have been discovered because of significant contribution of folklore knowledge of medicinal plants. There are enormous resources of medicinal plants mostly in developing countries.

*F. olivieri* belongs to the Zygophyllaceae family, which has about 25 genera and 240 species [11]. Regarding the medicinal uses *F. olivieri* is extensively in blood vascular, alimentary canal disorders, as antioxidant, analgesic, febrifuge, astringent and prophylactic against small-pox. This plant is also used for the treatment of cancer, asthma, urinary discharges, toothache, liver and kidney diseases in the indigenous system [12, 13]. Qualitative analysis of *F. olivieri* indicated the existence of saponins, terpenoids, alkaloids, flavonoids and cardiac glycosides [14]. *F. cretica* is locally used for liver troubles [15]. Thrombolytic activity has been reported for *F. arabica* [16]. Antipyretic activity in rat has been reported for *F. indica* [17]. Pareek et al. [18] investigated the i*n vitro* and in vivo antioxidant activity of *F. schweinfurthi* through DPPH, ABTS radicals and hydrogen peroxide methods and hepatoprotective potential against CCl_4_ induced toxicity in HepG2 cells and in rat. Hepatoprotective activity of *F. indica* has also been reported in earlier studies [19, 20]. Antitumor potential of *F. cretica* has been reported in different studies [21, 22].

The aims of our study were to determine the antioxidant activity of different fractions of methanol extract of *F. olivieri* compared to the quantitative content of total phenols and flavonoids in the extracts. The presence of the investigated phenolic components (quercetin, myricetin, kaempferol, catechin, gallic acid, caffeic acid, rutin and apigenin) in methanol extract was done by HPLC analysis. The methanol extract was also subjected to check its potential against acetaminophen induced hepatotoxicity in (*Rattus novergicus*) rats.

## Methods

### Chemicals

Sodium nitrite (NaNO_2_); sodium carbonate (Na_2_CO_3_); sodium hydroxide (NaOH); folin-ciocalteu reagent; 2, 2-diphenlyl-1-picrylhydrazyl (DPPH); aluminium chloride hexahydrate (AlCl_3_.6H_2_O); ferric chloride (FeCl_3_); phenazine methosulphate (PMS); nitro blue tetrazolium (NBT); trichloroacetic acid (TCA); thiobarbituric acid (TBA); ammoniummolybdate; hydrogen peroxide (H_2_O_2_); sodium phosphate; sulphuric acid; ethylenediaminetetraacetic acid (EDTA); 2, 2 azo bis, 3-ethylbenzothiozoline-6-sulphonic acid (ABTS); 2-deoxyribose; ascorbic acid; potassium ferricyanide [K_3_Fe (CN)_6_]; reduced glutathione (GSH); potassium persulfate (K_2_S_2_O_8_); bovine serum albumin (BSA) and 1,2-dithio-bis nitro benzoic acid (DTNB) were purchased from Sigma Co. (St. Louis, USA). All the solvents i.e. acetonitrile, methanol, acetic acid, dimethylsulfoxide (DMSO), hexane, chloroform, ethyl acetate and butanol were purchased from Sigma-Aldrich, Germany. Chemical standards for quercetin, myricetin, kaempferol, catechin, gallic acid, caffeic acid, rutin and apigenin were of analytical grade.

### Plant material

Aerial parts of *F. olivieri* were collected from Dhamyal Rawalpindi, Pakistan. Further identified by Dr. Saleem Ahmad and voucher specimen was deposited at Pakistan Museum of Natural History (Voucher No. 058608). Whole plant was collected and dried in shade for 1 month. Resulting dried sample was powdered using a blender and kept at room temperature in polythene bags.

### Extract preparation

Dried powdered sample (2 kg) of *F. olivieri* was extracted twice with 4 l of 95 % methanol at 40 °C for 7 days h and filtered. Filtrate was evaporated on rotary evaporator to get the crude extract (FOM). A part of crude methanol extract was suspended in distilled water (100 ml) and sequentially fractionated twice with n-hexane (FOH), chloroform (FOC) ethyl acetate (FOE), and n-butanol (FOB), however, the aqueous soluble portion was used as residual aqueous fraction (FOA), dried and was stored at 4 °C.

### Determination of total phenolic content

The total phenolics of the extract and various fractions of plant were determined by a reported method [23]. A volume of 200 μl (1 mg/ml of methanol) of the test sample was mixed with 1 ml of distilled water for dilution. Then 10 ml of 1:10 Folin-Ciocalteau reagent was added and the reaction misture was kept at 37 °C for 5 min. After incubation 7 ml of 0.115 mg/ml Na_2_CO_3_ was added in the mixture and kept again at 25 °C for for 2 h. The absorbance of the reaction mixture was recorded at 765 nm using a UV-visible spectrophotometer. The total phenolics are expressed as gallic acid equivalents (GAE) in miligram per gram (mg/g) of extract.

### Total flavonoid content estimation

Total flavonoid content of the extract/fractions was measured following the aluminium chloride colorimetric assay described earlier [24]. An aliquot of 0.25 ml of extract/fractions was combined with 1.25 ml of deionized H_2_O, then 75 μl of sodium nitrite (5 %) was added in the mixture. After 6 min 150 μl of AlCl_3_ (10 %) was mixed and incubated for 5 min with addition of 0.5 ml of 1 M NaOH. Absorbance was measured at 510 nm against a blank. The total flavonoid content was expressed as mg of rutin equivalents (RTE) per g of sample.

### HPLC analysis

Chromatographic analysis was carried out by using Agilent RP-C8 analytical column attached to HPLC-DAD. Mobile phase A was acetonitrile-methanol-water-acetic acid (5:10:85:1) and mobile phase B was acetonitrile-methanol-acetic acid (40:60:1). A gradient of time 0–20 min for 0 to 50 % B, 20–25 min for 50 to 100 % B and then 100 % B until 40 min. The flow rate was 1 ml/min and injection volume was 20 μl. Standards and plant extract stock solutions were prepared in methanol, at a concentration of 200 μg/ml and 10 mg/ml respectively. Samples were filtered through 0.45 μm membrane filter. Nine reference standards i.e. catechin, rutin, kaempferol, quercetin, gallic acid, salicylic acid, apigenin, myricetin and caffeic acid (Sigma company, USA) were run at appropriate wavelengths. Rutin and gallic acid were analyzed at 257 nm, catechin at 279 nm, caffeic acid at 325 nm and quercetin, myricetin, kampferol were analyzed at 368 nm. The column was reconditioned for 10 min before each run. All samples were assayed in triplicate. Quantification was carried out by the integration of the peak using the external standard method. All chromatographic operations were carried out at 20–25 °C temperature.

### In vitro antioxidant assays

The following antioxidant assays were performed on all the studied extract/fractions.

### DPPH (1, 1-diphenyl-2-picryl-hydrazyl) radical scavenging activity

The DPPH radical scavenging activity of extract/fractions was examined by comparison with that of a known antioxidant (ascorbic acid), using a reported method [25]. Different concentrations (250, 200, 150, 100, 50 and 25 μg/ml) of samples dissolved in methanol were taken in test tubes (100 μl) in triplicates. To prepare stock solution, 2.4 mg DPPH was dissolved in 100 ml of methanol. To attain an absorbance less than 1.00, the stock solution was further diluted with methanol. Then 3 ml of this solution was added to each test tube and shaken vigorously. After 30 min keeping at room temperature, absorbance was measured at 517 nm. The percent of DPPH decoloration of the samples was calculated according to the Eq. 1:

$$ \mathrm{DPPH}\kern0.5em \mathrm{scavenging}\kern0.5em \mathrm{activity}\ \left(\%\right)=\frac{\mathrm{Absorbance}\ \mathrm{of}\ \mathrm{control}-\mathrm{Absorbance}\ \mathrm{of}\ \mathrm{sample}}{\mathrm{Absorbance}\ \mathrm{of}\ \mathrm{control}}\times 100 $$

### Superoxide radical scavenging assay

The scavenging activity of the extract/fractions against superoxide anion was determined according to the method of Ponti et al. [26]. The reaction mixture containing 250 μl of potassium phosphate buffer (0.05 M; pH 7.6), phenazine methosulphate (0.02 M, 126 μl), nitroblue tetrazolium (0.005 M, 50 μl) and riboflavin (0.05 M, 150 μl) was mixed with 150 μl of test sample (1 mg/ml methanol) at different concentrations (250, 200, 150, 100, 50 and 25 μg/ml in methanol). The mixture was placed under the fluorescent lamp for 20 min to initiate the reaction. Absorbance was recorded at 560 nm against a blank. Ascorbic acid was used as standard. The scavenging effect was determinted by using Eq. 1.

### Phosphomolybdate assay (total antioxidant capacity)

To estimate the scavenging abilities of the plant samples phosphomolybdenum assay was conducted according to Prieto et al. [27]. Serial dilutions of the plant samples were made in DMSO. The reaction mixture was prepared by mixing of 5 ml of 28 mM Na_3_PO_4_ and 0.6 M H_2_SO_4_ with that of 4 mM ammonium molybdate. From the sample solutions 0.2 ml was mixed with 2 ml of the reaction mixture and incubated in a water bath at 90 °C for 80 min. After cooling to room temperature the absorbance was recorded at 765 nm against blank. The antioxidant capacity was calculated by using Eq. 1.

### ABTS (2,2′-azino-bis(3-ethylbenzothiazoline-6-sulphonic acid) scavenging activity

The ABTS scavenging ability of the extracts was determined according to the method described by Miller et al. [28]. The ABTS^•+^ (cation) was generated freshly by mixing the ABTS solution (7 mM) with 2.45 mM K_2_S_2_O_8_ and kept in dark for 8 h. The absorbance of the ABTS was adjusted to 0.7 after measuring at 745 nm by the addition of ethanol (70 %). Aliquot of 100 μl of various concentrations (250, 200, 150, 100, 50 and 25 μg/ml in methanol) were added in 1 ml of ABTS solution. Alteration in absorbance was recorded for 6 min. By the use of Eq. 1, ABTS cation scavenging potential of plant extract was determined.

### Hydroxyl radical scavenging activity

The hydroxyl radical scavenging activity was determined according to the method of Halliwell et al. [29]. DMSO was used as the solvent to dissolve and for serial dilution of the plant samples. The reaction mixture was prepared by the addition of 75 μl of different concentrations of the extracts with 450 μl of sodium phosphate buffer (0.2 M, pH 7.0), 150 μl of 2 deoxyribose (10 mM), 150 μl of FeSO_4_-EDTA (10 mM), 150 μl of H_2_O_2_ (10 mM) and 525 μl of H_2_O. After incubation at 37 °C for 4 h, the reaction was stopped by the addition of 750 μl of trichloroacetic acid (2.8 %). After that 750 μl of thiobarbituric acid (1 %) in 50 mM NaOH was added and the reaction mixture was heated in water bath for 10 min. Absorbance of the reaction mixture was recorded at 520 nm once the mixture temperature fall to room temperature. Ascorbic acid served as standard and scavenging activity was estimated by Eq. 2.

$$ \mathrm{Scavenging}\ \mathrm{activity}\ \left(\%\right)=\frac{1-\mathrm{Absorbance}\ \mathrm{of}\ \mathrm{sample}}{\mathrm{Absorbance}\ \mathrm{of}\ \mathrm{control}} \times 100 $$

### Hydrogen peroxide scavenging activity

Hydrogen peroxide scavenging activity of the plant extract/fractions was determined by the method of Ruch et al. [30]. Serial dilutions of the plant samples were prepared in concentration of 250, 200, 150, 100, 50 and 25 μg/ml in methanol. Then 100 μl of each concentration was added to 300 μl of 50 mM phosphate buffer (pH 7.4) followed by the addition of 0.6 ml of 2 mM H_2_O_2_ solution and kept at 37 °C for 15 min. The absorbance of the reaction mixture was recorded at 230 nm against blank. Hydrogen peroxide scavenging ability (in triplicate) was calculated by following Eq. 2:

### Reducing power

The reducing power of the extract/fractions was determined according to Landry et al. [31]. An aliquot of 100 μl of different concentrations (250, 200, 150, 100, 50 and 25 μg/ml) were mixed with 100 μl of 0.3 M phosphate buffer-(pH 6.8) and 100 μl of-potassium ferricyanide (10 mg/l) and the reaction cocktail was placed in the incubator for 20 min at 80 °C for 10 min. Then 250 μl of 10 % trichloroacetic acid was added to the mixture, which was then centrifuged at 3000 rpm for 10 min. The supernatant (0.25 ml) was mixed with 0.25 ml of distilled water and 0.5 ml of ferric chloride solution (0.1 %). The absorbance of the reaction mixture was recorded at 700 nm. Increased absorbance of the reaction mixture indicated a high reducing power.

### In vivo acetaminophen induced hepatotoxicity in rat

#### Animals

Six weak old male Sprague–Dawley rats weighing 180–200 g were housed in conventional cages and maintained at 24 ± 3 °C under 12 h light/dark cycle at Primate Facility of the Quaid-i-Azam University Islamabad, Pakistan. The animals were allowed free access to standard basal and water ad libitum. The basal diet was composed of 20 % protein (casein), 10 % sucrose, 5 % corn oil, 2 % choline chloride, 1 % vitamin mixture, 3.5 % salt mixture and 5 % fibers (cellulose). The remainder was corn starch up to 100 %. The use of animals in experiment was approved (Bch#245) by Ethical Committee of Quaid-i-Azam University, Islamabad and conducted in accordance with guidelines established by the National Institute of Health (NIH guidelines Islamabad, Pakistan).

#### Acute toxicity test

Acute toxicity of the extract was assessed by the guidelines 425 advocated by the Organization for Economic Cooperation and Development (OECD) [32]. The animals used for acute toxicity test were kept in fasting conditions for overnight with just water availability. The plant samples were prepared in DMSO and three animals were intra-gastrically administered with dose of 50 mg/kg bw and were monitored for mortality for 2 weeks. After initial screening of toxicity higher doses of the extract/fractions i.e., 100, 200, 400, 1000, 2000, 3000 and 4000 mg/kg bw were administered to three rats for each treatment. At these administered doses mortality and abnormal behaviour was not noticed, so one tenth of the highest dose (400 mg/kg) and 200 mg/kg were selected for the evaluation of hepato-protective propagation activities.

#### Experimental protocol

To assess the hepatoprotective potential of the crude methanol extract of *F. olivieri* the Sprague–Dawley (*Rattus novergicus*) male rats were randomly divided into six groups with six rats in each. Group-I: served as normal control and received 1 ml of saline (0.9 %). Group-II: animals were administered intragastrically 750 ml/kg of acetaminophen for 7 days [33].

Group-III: animals of this group received acetaminophen as well as 50 mg/kg of the standard drug silymarin. Group-IV and V: animals of these two groups received acetaminophen along with methanol extract of *F. olivieri* at 200 mg/kg and 400 mg/kg respectively. However, animals of Group-VI received only 400 mg/kg of *F. olivieri* methanol extract. All the treatments were administered intragastrically through feeding tube in the morning. Animals were euthanized after light ether anesthesia after 24 h of the last treatment. The blood was collected by cardiac puncture into plain tubes and stored at 4 °C.

#### Body weight and organ weight

Body weight of each animal in a specific group was recorded before and after the experimentation. On the basis of initial and final weight percent increase in body weight was estimated. After euthesia liver was removed, rinsed in cold saline, weighted and relative liver weight was determined for each animal of a group.

#### Haematological studies

Various haematological parameters such as percent of packed cell volume (PCV), haemoglobin (g/l), mean corpuscular volume (MCV) in femtolitres (fL), mean corpuscular haemoglobin concentration (MCHC, g/dl), mean corpuscular haemoglobin test (MCH; pg) and count of whilte blood cells (WBC, 10^3^/μl), total leukocyte count (TLC × 10^3^/μl), platelet count (PLT × 10^3^/μl), lymphocytes (%), neutrophils (%) and granulocytes (%) were estimated through the cell dyn ruby automated 5 part hematology analyser (Abbott diagnostics, Germany).

#### Blood serum studies

We have estimated the level of liver markers enzymes (alanine aminotransferase (ALT), aspartate aminotransferase (AST), alkaline phosphatase (ALP), lactate dehydrogenase (LDH), total bilirubin and protein, lipid profile (total cholesterol, low-density lipoprotein (LDL), high-density lipoprotein (HDL) and triglyceride by using standard AMP diagnostic kits (Stattogger Strasse 31b 8045 Graz, Austria).

#### Assessment of oxidative stress

Liver tissue homogenates were prepared in 10 volume of 100 mM KH_2_PO_4_ buffer containing 1 mM EDTA (pH 7.4) and centrifuged at 12,000 × g for 30 min at 4 °C. The supernatant was collected and used for the estimation of oxidative stress. The concentration of protein in the supernatant of hepatic homogenate was assessed using crystalline BSA as standard.

#### Catalase assay (CAT)

Catalase activities in the supernatant obtained from liver homogenates were estimated by the method of Chance and Maehly [34]. The reaction mixture for CAT activity was prepared by the addition of 2.5 ml of 50 mM phosphate buffer (pH 5.0), 0.4 ml of 5.9 mM H_2_O_2_ and 0.1 ml of the supernatant. Changes in absorbance of the reaction solution at 240 nm were determined for 1 min. One unit of CAT activity was defined as an absorbance change of 0.01/min. The results are expressed in units/mg protein.

#### Superoxide dismutase assay (SOD)

In the liver homogenates of various groups SOD activity was estimated by the method of Kakkar et al. [35]. To concentrate the superoxide dismutase enzyme in the upper layer of supernatant the liver homogenate was centrifuged at 10,000 *g* for 15 min. A volume of 150 μl of the supernatant was added to the reaction mixture containing 0.6 μl of sodium pyrophosphate buffer (0.052 mM, pH 7.0) and 50 μl of phenazine methosulphate (186 μM). Finally, 100 μl of NADH (780 μM) was added to initiate enzymatic reaction. Glacial acetic acid (0.5 ml) after 1 min was added in the reaction mixture to stop the reaction. Absorbance was determined at 560 nm to quantify the color intensity. Results were expressed in units/mg protein.

#### Glutathione reductase assay (GR)

Glutathione reductase activity was determined by following the method of Carlberg and Mannervik [36]. Liver supernatant 0.1 ml was added in the reaction mixture consisted of 1.65 ml phosphate buffer (0.1 M; pH 7.6), 0.1 ml of EDTA (0.5 mM), 0.05 ml of oxidized glutathione (1 mM) and 0.1 ml of NADPH (0.1 mM). Decomposition of NADPH was measured spectrophotometrically at 340 nm at 25 °C. GR activity was determined by multiplying the absorbance value obtained with molar extinction coefficient of 6.23 × 10^3^/M/cm and were expressed as amount of NADPH oxidized/min/mg protein.

#### Glutathione peroxidase assay (GSH-Px)

Glutathione peroxidase activity was assayed by the method of Mohandas et al. [37]. An aliquot of 0.1 ml of the hepatic supernatant was mixed with the reaction mixture consisted of 1.49 ml of phosphate buffer (0.1 M; pH 7.4), 0.1 ml of EDTA (1 mM), 0.1 ml of sodium azide (1 mM), 0.05 ml of glutathione reductase (1 IU/ml), 0.05 ml of GSH (1 mM), 0.1 ml of NADPH (0.2 mM) and 0.01 ml of H_2_O_2_ (0.25 mM). Oxidation of substrate i.e. NADPH was determined at 340 nm spectrophotometrically. Activity GSH-Px was calculated as amount of NADPH oxidized per min per mg protein with the aid of molar extinction coefficient of 6.23 × 10^3^/M/cm.

#### Reduced glutathione assay (GSH)

Reduced glutathione was estimated by the method of Jollow et al. [38]. To estimate the concentration of GSH in the samples 1.0 ml of liver supernatant was precipitated with 1.0 ml of (4 %) sulfosalicylic acid. The samples were kept at 4 °C for 1 h and then centrifuged at 1200 *g* for 20 min at 4 °C. Then from the supernatant 0.1 ml was added in the mixture consisted of 2.7 ml of phosphate buffer (0.1 M; pH 7.4) and 0.2 ml of dithio-bis nitro benzoic acid (DTNB, 100 mM). GSH reacts with DTNB and produces a yellow colored complex. Absorbance was measured at 412 nm. It was expressed as μM GSH/g tissue.

#### Estimation of lipid peroxidation assay (TBARS)

The assay for lipid peroxidation was carried out following the method of Iqbal et al. [39]. In order to assess the concentration of lipid peroxides in the liver samples; 0.2 ml of the supernatant was added in the reaction mixture having 0.58 ml of phosphate buffer (0.1 M; pH 7.4), 0.2 ml of ascorbic acid (100 mM), and 0.02 ml of ferric chloride (100 mM). The reaction mixture was incubated at 37 °C in a shaking water bath for 1 h. The reaction was stopped by addition of 1.0 ml of 10 % trichloroacetic acid. Following addition of 1.0 ml of 0.67 % thiobarbituric acid, all the tubes were placed in boiling water bath for 20 min and then shifted to crushed ice-bath to cool down the mixture. It was centrifuged at 2500 *g* for 10 min and absorbance of the supernatant was recorded at 535 nm against a reagent blank. The amount of thiobarbituric acid reactive substances (TBARS) formed in each of the samples was assessed by measuring optical density of the supernatant at 535 nm using spectrophotometer against a reagent blank. The results were expressed as nM TBARS/min/mg tissue at 37 °C using molar extinction coefficient of 1.56 × 10^5^ M^−1^cm^−1^.

#### DNA damaging studies

DNA damaging effects of the paracetamol in liver samples were assessed following the procedure of Wu et al. [40]. Hepatic tissue (100 mg) was homogenized in 10 volume of a solution consisting of 5 mM Tris–HCl, 20 mM EDTA (pH 8.0) and 0.2 % triton X-100. A volume of 1.0 ml of each sample was centrifuged at 27,000 *g* at 4 °C for 20 min. The pellet obtained was labelled B represent the intact chromatin whereas chromatin of the supernatant represented the fragmented and labelled T. Supernatant and the pellet were assayed for the assessment of DNA contents using a freshly prepared diphenylamine (DPA) solution reaction. Absorbance of reaction mixture was recorded at 600 nm with a UV/VIS spectrophotometer. The results were expressed as amount of % fragmented DNA by the following formula:

$$ \mathrm{Fragmented}\ \mathrm{D}\mathrm{N}\mathrm{A}\ \left(\%\right)=\frac{\mathrm{T}}{\mathrm{T}+\mathrm{B}} \times 100 $$

#### Histopathological studies

For histopathological evaluation a portion of liver was fixed in a fixative (absolute alcohol 60 %, formaldehyde 30 %, acetic acid 10 %). The tissues were embedded in paraffin, sectioned at 4 μm and subsequently stained with hematoxylin/eosin. Sections were studied under light microscope (DIALUX 20 EB) at 40 and 100 magnifications. Slides of all treated groups were studied and photographed.

#### Statistical analysis

The values were expressed as mean ± standard error. For in vivo studies, the consequences of different treatments given to animals were evaluated by one way analysis of variance which was carried by means of computer software GraphPad prism 4.0. Multiple comparisons among various treatments were made by least significance difference (LSD) method at *p*-value ≤ 0.05. Linear regression between the IC_50_ values of various assays and the total phenolic and total flavonoid content were computed at *p* <0.05 and *p* <0.01.

## Results

### Extraction yield

The extraction yields of different fractions are given in Table 1. The yield of the methanol extract 12.56 % to that of the dry powder was recorded. Among the fractions the aqueous fraction has the maximum yield of 10.59 %, n-butanol 9.025 %, ethyl acetate 8.25 %, n-hexane 2.21 % and the chloroform fraction with 1.98 % yield to that of the methanol extract.

**Table 1:** Total phenolic and flavonoid content and extraction yield of *F. olivieri* fractions

	Total phenolics (mg gallic acid equivalent/g)	Total flavonoids (mg rutin equivalent/g)	Extraction yield (%)
Methanol extract	32 ± 1.202^e^	16 ± 0.881^e^	12.56
n-Hexane fraction	0.00	0.00	2.21
Chloroform fraction	44 ± 1.263^d^	26 ± 0.666^d^	1.98
Ethyl acetate fraction	106 ± 0.892^a^	40 ± 1.155^b^	8.25
n-Butanol fraction	48 ± 2.517^c^	32 ± 0.873^c^	9.025
Aqueous fraction	66 ± 0.333^b^	50 ± 1.764^a^	10.59

Mean ± SE (*n* = 3). Means not sharing the same letter (^a-e^) are significantly different (LSD) at *p* < 0.01 probability level in each column. Crude methanol extract yield was based on the dry weight of powder whereas the fractions yield was based on the dry weight of crude methanol extract

### Total phenolic content

Total phenolic content as measured by folin-Ciocalteu method, werre reported as gallic acid equivalents (GAE) are presented in Table 1. The highest amount of phenolic content was found in the aqueous fraction (50 ± 1.764 mg GAE/g extract), while the least amount was observed in the methanol extract (16 ± 0.881 mg/g GAE). Presence of phenolic contents was not indicated in the n-hexane fraction (Table 1).

### Total flavonoid content

Total flavonoid contents of different fractions are also shown in Table 1. The flavonoid contents of the extracts in terms of rutin equivalent (RTE) were between 19 ± 0.529 and 106 ± 0.892 mg/g extract with the descending order of ethyl acetate > aqueous > n-butanol > chloroform > methanol. The occurrence of flavonoid contents was not indicated in the n-hexane fraction.

### HPLC analysis of nethanol extract

**Fig. 1:**
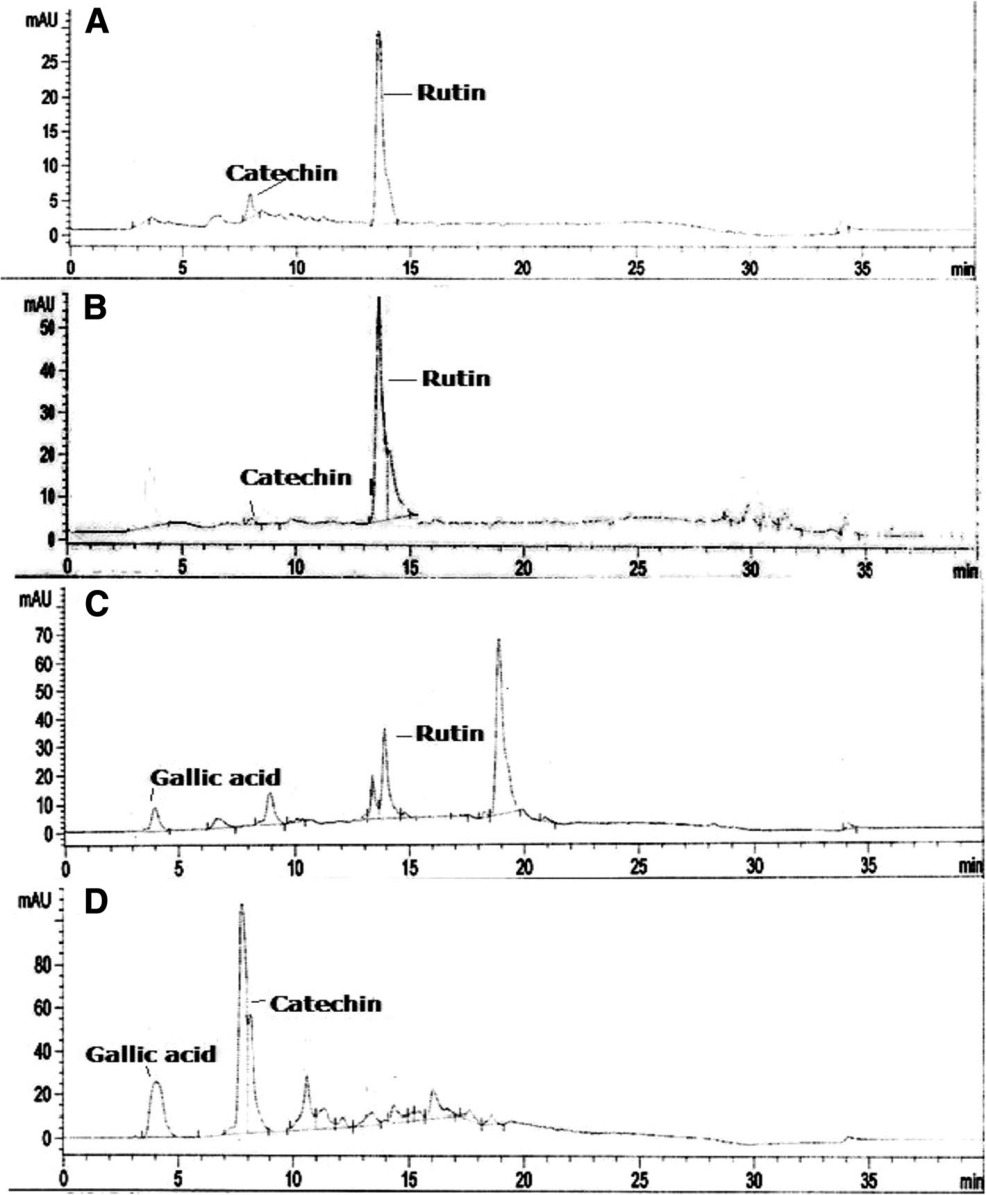
HPLC fingerprint of methanol extract of *F. olivieri* and its derived fractions. (a) *FOM*, *F. olivieri* methanol extract; (b) *FOE*, *F. olivieri* ethyl acetate fraction of FOM; (c) *FOB*, *F. olivieri* n-butanol fraction of FOM; (d) *FOA*, *F. olivieri* soluble residual aqueous fraction of FOM

In this study, nine phenolic compounds have been investigated in *F. olivieri* extract and the chromatographic profiles of polyphenolics is presented in Table 2. The HPLC fingerprinting of *F. olivieri* extract revealed the presence of gallic acid, caffeic acid, rutin, myricetin and catechin (Fig. 1). These polyphenols were identified by comparing the retention times and UV spectra to authentic standards analyzed under identical analytical conditions. Among the polyphenolics rutin was recorded in methanol extract (2.831 μg/mg dry weight), ethyl acetate fraction (2.145 μg/mg dry weight) and in n-butanol fraction (0.544 μg/mg dry weight). Catechin showed its presence in methanol extract (0.101 μg/mg dry weight), ethyl acetate (0.0768 μg/mg dry weight) and in aqueous fraction (0.824 μg/mg dry weight). The HPLC fingerprints revealed the existence of gallic acid in n-butanol (0.049 ± 0.002 μg/mg dry weight) and aqueous fraction (0.529 μg/mg dry weight).

**Table 2:** Polyphenolics composition of *F. olivieri* fractions by HPLC

Plant extracts	Phenolic compounds	Retention Time (min)	Wavelength (nm)	Concentration (μg/mg dry weight)
FOM	Catechin	8.122	279	0.101
	Rutin	14.349	257	2.831
FOE	Catechin	8.122	279	0.076
	Rutin	14.349	257	2.145
FOB	Gallic acid	4.4	257	0.049
	Rutin	14.349	257	0.544
FOA	Gallic acid	4.4	257	0.529
	Catechin	8.122	279	0.824

### In vitro antioxidant studies

#### DPPH scavenging activity

**Fig. 2:**
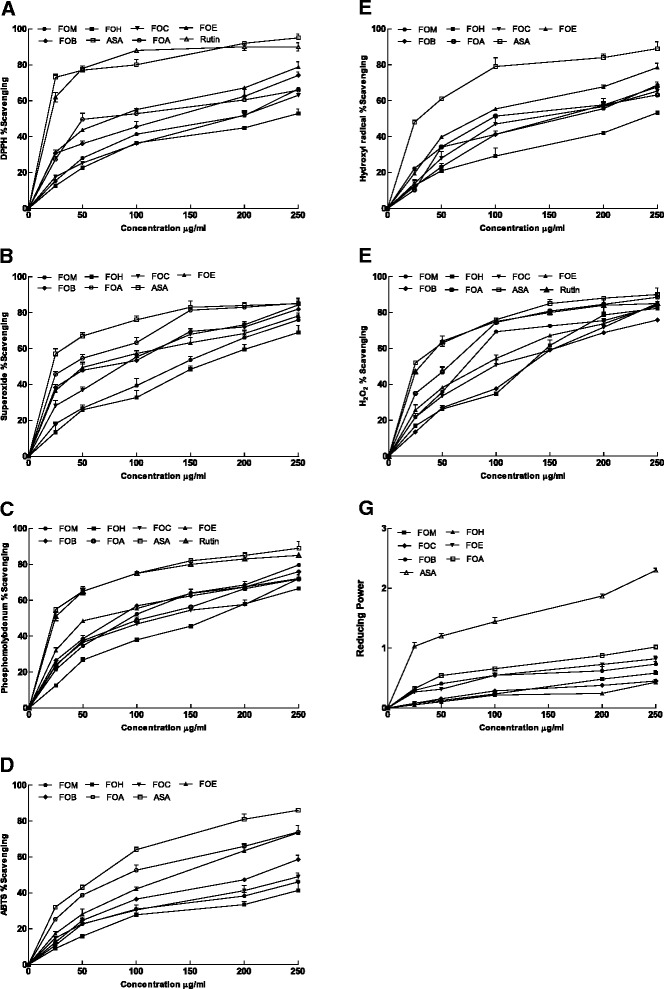
Radical scavenging activity of different fractions of *F. olivieri* at different concentrations. (**a**) DPPH; (**b**) superoxide; (**c**) phosphomolybdenum; (**d**) ABTS; (**e**) hydroxyl; (**f**) H_2_O_2_; (**g**) reducing power. Each value represents a mean ± SEM (*n* = 3). *FOM*, *F. olivieri* methanol extract; *FOH*, *F. olivieri* n-hexane fraction of FOM; *FOC*, *F. olivieri* chloroform fraction of FOM; *FOE*, *F. olivieri* ethyl acetate fraction of FOM; *FOB*, *F. olivieri* n-butanol fraction of FOM; *FOA*, *F. olivieri* soluble residual aqueous fraction of FOM; *ASA*, ascorbic acid

The 50 % scavenging of DPPH radical (IC_50_) of the methanol extract and its different fractions is presented in Table 3. A lower IC_50_ value would reflect greater antioxidant activity of the sample. In the present study the highest radical scavenging activity was shown by the aqueous extract (IC_50_ = 55 ± 1.212 μg/ml), whereas the n-hexane extract showed lowest activity (IC_50_ = 227 ± 1.0 μg/ml). The extract and fractions exhibited dose dependent scavenging of DPPH (Fig. 2).

**Table 3:** Antioxidant effect *F. olivieri* on various antioxidant assays

Plant extract	IC_50_ μg/ml					
	Scavenging ability on DPPH radicals	Scavenging ability on superoxide radicals	Phospho- molybdate assay	Scavenging ability on hydroxyl radicals	Scavenging ability on hydrogen peroxide	Scavenging ability on ABTS radicals
FOM	183 ± 1.528^d^	137.8 ± 2.33^e^	99 ± 2.028^c^	162 ± 2.404^e^	71 ± 1.202^b^	269 ± 1.525^e^
FOH	227 ± 1.0^f^	159 ± 1.475^f^	167 ± 1.732^f^	235 ± 1.743^f^	125 ± 0.819^f^	>300^f^
FOC	187 ± 1.453^e^	85.8 ± 1.091^d^	124 ± 1.284^e^	125 ± 1.451^c^	96 ± 0.577^d^	255 ± 2.021^d^
FOE	76.5 ± 1.014^c^	53.1 ± 1.721^c^	78 ± 0.883^b^	82 ± 2.603^b^	83 ± 1.473^c^	138 ± 1.736^c^
FOB	132 ± 2.082^d^	68.5 ± 1.200^d^	83 ± 1.856^c^	154 ± 0.881^d^	117 ± 1.155^e^	213 ± 1.458^d^
FOA	55.7 ± 1.212^b^	37.1 ± 0.643^b^	112 ± 2.414^d^	91 ± 2.186^b^	64 ± 1.463^b^	90 ± 1.232^b^
ASA	16.5 ± 0.82^a^	21.8 ± 1.36^a^	22.5 ± 0.41^a^	29.1 ± 0.34^a^	23.8 ± 1.21^a^	66.5 ± 0.74^a^
Rutin	19.8 ± 1.12^a^	-	25.8 ± 2.62^a^	-	28.5 ± 1.26^a^	-

Mean ± SE (*n* = 3). Means not sharing the same letter (^a-f^) are significantly different (LSD) at *p* < 0.05 probability level in each column

#### Superoxide radical scavenging activity

The superoxide radical scavenging activity of the extract/fractions is presented in Table 2, as determined in vitro by the riboflavin-NBT-light system. The highest scavenging activity was determined in aqueous extract (IC_50_ = 37.1 ± 0.643 μg/ml). The methanol extract, ethyl acetate, chloroform and *n*-butanol fraction exhibited IC_50_ value of 137.8 ± 2.339, 53.1 ± 1.721, 85.8 ± 1.091 and 68.5 ± 1.200 μg/ml, respectively. *n*-Hexane fraction exhibited the lowest superoxide radical scavenging activity with IC_50_ value of 159 ± 1.475 μg/ml (Table 3). The extract/frcations exhibited dose dependent response for superoxide radical scavenging activity (Fig. 2).

#### Phosphomolybdenum assay

Phosphomolybdenum assay was used to determine the antioxidant capacity of the extract/fractions of *F. olivieri*. It relies on Mo (IV) reduction to Mo (V) by the sample analyte, leading to the formation of green phosphate/Mo (V) compounds with absorption at 695 nm. The extract/fractions were found to have IC_50_ values that decreased in the order; ethyl acetate > n-butanol > methanol > aqueous > chloroform > n-hexane (Table 3). There was an increase in antioxidant capacity in all the extract/fractios with increase in dose (Fig. 2).

#### Hydroxyl radical scavenging potential

The highest hydroxyl radical scavenging potential was shown by ethyl acetate fraction (IC_50_ = 82 ± 2.603 μg/ml) followed by aqueous (IC_50_ = 91 ± 2.186 μg/ml), chloroform (IC_50_ = 125 ± 1.451 μg/ml), n-butanol (IC_50_ = 154 ± 0.881 μg/ml), methanol (IC_50_ = 162 ± 2.404 μg/ml) and n-hexane (IC_50_ = 235 ± 1.743 μg/ml). The hydroxyl radical scavenging activity of the crude methanol extract/fractions of *F. olivieri* exhibited dose response activity (Fig. 2).

#### Hydrogen peroxide scavenging activity

Scavenging activity of hydrogen peroxide shown by different extract/frcations of *F. olivieri* are as follows: methanol (IC_50_ = 71 ± 1.202 μg/ml), n-hexane (IC_50_ = 125 ± 0.819 μg/ml), chloroform (IC_50_ = 96 ± 0.577 μg/ml), ethyl acetate (IC_50_ = 83 ± 1.473 μg/ml), n-butanol (IC_50_ = 117 ± 1.155 μg/ml) and aqueous (IC_50_ = 64 ± 1.463 μg/ml) (Table 3). A concentration dependent increase in scavenging potential of the extract/fractions was observed (Fig. 2).

#### ABTS radical scavenging activity

The scavenging activity of plant extract/frcations against ABTS radical cation is presented in Table 3. The aqueous extract exhibited the highest value (IC_50_ = 90 ± 1.232 μg/ml) followed by ethyl acetate (IC_50_ = 138 ± 1.736 μg/ml), n-butanol (IC_50_ = 213 ± 1.458 μg/ml), chloroform (IC_50_ = 255 ± 2.021 μg/ml), methanol (IC_50_ = 269 ± 1.525 μg/ml) and n-hexane (>300). All the extrac/fractions exhibited dose response scavenging potential towards the ABTS radicals (Fig. 2).

#### Reducing power assay

The reducing ability of the extract/fractions of *F. olivieri* exhibited a descending trend with aqueous > ethyl acetate *> n-*butanol > methanol > chloroform > *n*-hexane (Fig. 2).

#### Correlation studies

The total flavonoid contents were significantly correlated; R^2^ = 0.9413, R^2^ = 0.8551 and R^2^ = 0.8130 with the antioxidant activities for ABTS, DPPH and the superoxide radicals respectively (Table 4). However, low level of correlation was noticed with hydroxyl radical, hydrogen peroxide and phosphomolybdenum assay. Total phenolic content exhibited moderate level of association R^2^ = 0.7329; *p* <0.05, R^2^ = 0.7100; *p* < 0.05 for hydroxyl and DPPH radicals respectively. For the rest of antioxidant assays medium to low level of association were recorded for superoxide radical, ABTS radical, phosphomolybdate and for hydrogen peroxide scavenging respectively (Table 4).

**Table 4:** Correlation between antioxidant activities and total phenolic and flavonoid content

	Correlation R^2^	
IC_50_ values	Phenolics	Flavonoids
IC_50_ of scavenging ability on DPPH radicals	0.7100^a^	0.8551^b^
IC_50_ of scavenging ability on superoxide radicals	0.6253	0.8130^a^
IC_50_ of phosphomolybdate assay	0.4562	0.1339
IC_50_ of scavenging ability on hydroxyl radicals	0.7329^a^	0.5616
IC_50_ of scavenging ability on hydrogen peroxide	0.1991	0.1610
IC_50_ of scavenging ability on ABTS radicals	0.5373	0.9413^b^

^a, b^ Indicate significance at *p* <0.05 and *p* <0.01 respectively

### Animal studies

#### Acute toxicity studies

Actute toxicity results of this study indicated that the crude methanol extract of *F. olivieri* did not cause mortality even at its highest dose of 4000 mg/kg to rats. The abnormal behaviour was not observed for 14 days after the administration of various doses of methanol extract to rats. On the basis of these results 400 mg/kg dose was used as the highest dose for animal treatment.

#### Effects of *F. olivieri* on body and liver weight

The protective effects of *F. olivieri* against acetaminophen induced variations in the body weights are presented in Table 5. There was a decline in percent increase of body weights in rats of acetaminophen-treated group (750 mg/kg) as compared to the animals of the control group. Co-administration of the rats with acetaminophen and the methanol extract of *F. olivieri* considerably (*p* <0.05) reversed the changes in body weights of rats towards the control animals. Similar effects were studied on the body weights of rats treated with silymarin and were comparable to the higher doses (400 mg/kg) of *F. olivieri*. Administration of *F. olivieri* alone to rats was unable (*p* >0.05) to change the body weights as compared to the control rats.

**Table 5:** Effect of FOM body weight and liver weight in acetaminophen treated rats

	Initial body weight (g)	Final body weight (g)	Percent increase in body weight	Absolute liver weight (g)	Relative liver weight (% to body weight)
Control (Saline)	152.82 ± 2.16	214.83 ± 2.21	40.57 ± 2.34^d^	7.78 ± 1.84^c^	0.077 ± 0.0003^d^
AMP 750	153.33 ± 1.60	197.62 ± 4.85	28.88 ± 1.53^a^	3.08 ± 0.53^a^	0.030 ± 0.0001^a^
AMP + Sily 50	156.00 ± 3.00	215.50 ± 1.88	38.14 ± 2.26^c^	7.53 ± 1.62^c^	0.075 ± 0.0004^c^
AMP + FOM 200	154.83 ± 1.24	204.23 ± 2.80	31.90 ± 1.75^b^	5.39 ± 0.25^b^	0.053 ± 0.0007^b^
AMP + FOM 400	153.33 ± 1.56	210.67 ± 1.65	37.39 ± 2.92^c^	7.56 ± 0.38^c^	0.075 ± 0.0002^c^
FOM 400	158.67 ± 1.18	220.16 ± 1.12	38.75 ± 1.64^c^	7.84 ± 1.57^c^	0.078 ± 0.0001^d^

Mean ± SEM (06). Means with different superscript letters (^a-d^) within the column indicate significant difference (*p* <0.05). *AMP*, acetaminophen; *FOM*, *F. olivieri* methanol extract

A considerable decline was observed in the absolute and relative weights of liver of acetaminophen treated rats as compared to control group (*p* < 0.05). Crude methanol extract at 200 mg/kg and 400 mg/kg co-administration maintained the organ weight towards the control group (Table 5). Similar, results were also observed with the co-administration of silymarin (50 mg/kg) for the absolute and the relative weights of liver. Treatment of rats with the methanol extract alone in this experiment did not decrease the liver weights as against the control group.

#### Effects of *F. olivieri* on haematological parameters

A significant decline was observed in the values of PCV, Hb, MCHC and MCH while increase in MCV values in rats after acetaminophen administration (*p* <0.05) as compared to the control group. Co-administration of both the doses of *F. olivieri* (200 and 400 mg/kg) with acetaminophen were able to revert these values towards the control level (Table 6). Administration of *F. olivieri* methanol extract (400 mg/kg) alone showed non significant (*p* > 0.05) alterations as compared to the control group. However, silymarin co-administration (50 mg/kg) with acetaminophen considerably (*p* <0.05) removed the acetaminophen toxicity and restored the values of PCV, Hb, MCHC, MCV and MCH towards the control rats.

**Table 6:** Effect of FOM blood parameters in acetaminophen induced toxicity in rat

Treatment (mg/kg bw)	PCV (%)	Hb (g/l)	MCV (fL)	MCHC (g/dl)	MCH (pg)
Control (Saline)	37.23 ± 2.13^d^	13.35 ± 0.32^e^	60.23 ± 1.50^d^	33.73 ± 1.25^d^	18.62 ± 1.28^c^
AMP 750	32.15 ± 2.01^a^	8.19 ± 0.63^a^	64.47 ± 2.55^a^	28.38 ± 0.52^a^	15.22 ± 1.67^a^
AMP + Sily 50	37.11 ± 1.15^d^	12.93 ± 0.31^d^	61.13 ± 1.42^c^	33.19 ± 1.51^d^	18.14 ± 1.11^c^
AMP + FOM 200	34.44 ± 1.27^b^	9.74 ± 0.16^b^	63.80 ± 2.13^b^	31.19 ± 1.52^b^	16.15 ± 0.90^b^
AMP + FOM 400	36.32 ± 2.05^c^	10.32 ± 0.42^c^	61.69 ± 3.82^c^	32.62 ± 1.59^c^	18.19 ± 2.50^c^
FOM 400	38.01 ± 2.63^e^	12.17 ± 0.15^d^	59.22 ± 2.41^e^	34.85 ± 0.84^e^	19.25 ± 2.16^d^

Mean ± SEM (06). Different superscript letters (^a-e^) within the column indicate significant difference (*p* <0.05). *AMP*, acetaminophen; *FOM*, *F. olivieri* methanol extract

After acetaminophen treatment, the values of WBC and neutrophil count were decreased significantly (*p* <0.05) in comparison to control animals. In contrast, TLC, PLT, lymphocytes and granulocytes count elevated in acetaminophen treated animals when compared with control group. The values of WBC and neutrophils increased significantly (*p* <0.05) in the *F. olivieri* extract co-treated groups (200 and 400 mg/kg), while significant (*p* <0.05) decrease in the count of TLC, PLT, lymphocytes and granulocytes was recorded (Table 7). Co-treatment of silymarin (50 mg/kg) with acetaminophen showed similar ameliorating effects in rats. There was not any significant change in the count of WBC, neutrophils, TLC, PLT, lymphocytes and granulocytes in the rats which were given 400 mg/kg of *F. olivieri* extract alone (Table 7).

**Table 7:** Effect of FOM on blood parameters in acetaminophen induced toxicity in rat

Treatment (mg/kg bw)	WBC (10^3^/μl)	TLC (× 10^3^/μl)	PLT (× 10^3^/μl)	Lymphocytes (%)	Neutrophils (%)	Granulocytes (%)
Control (Saline)	7.16 ± 0.20^b^	5.31 ± 0.47^c^	710.16 ± 10.64^c^	37.15 ± 3.45^c^	60.15 ± 3.20^c^	9.57 ± 0.52^e^
AMP 750	5.62 ± 0.22^a^	7.62 ± 0.25^a^	865.21 ± 118.32^a^	59.43 ± 1.53^a^	35.52 ± 1.21^a^	15.12 ± 2.01^a^
AMP + Sily 50	7.64 ± 0.14^d^	5.11 ± 0.43^c^	702.19 ± 4.01^c^	40.75 ± 2.15^c^	62.83 ± 0.52^c^	11.77 ± 0.84^d^
AMP + FOM 200	7.44 ± 0.45^c^	6.51 ± 0.75^b^	706.51 ± 56.36^c^	47.18 ± 2.11^b^	41.38 ± 1.90^b^	11.29 ± 2.18^d^
AMP + FOM 400	7.82 ± 0.54^d^	5.63 ± 0.12^c^	843.15 ± 23.17^b^	39.26 ± 1.22^c^	58.71 ± 0.95^c^	14.53 ± 0.33^b^
FOM 400	7.58 ± 0.29^c^	5.10 ± 0.19^c^	708.16 ± 7.89^c^	34.11 ± 1.28^c^	63.43 ± 1.18^c^	12.93 ± 0.31^c^

Mean ± SEM (06). Means with different superscript letters (^a-e^) within the column indicate significant difference (*p* <0.05). *AMP*, acetaminophen; *FOM*, *F. olivieri* methanol extract

#### Effects of *F. olivieri* on liver marker enzymes

The values for liver marker enzymes for acetaminophen, silymarin, *F. olivieri* treated rats and control group are depicted in Table 8. The values of ALT, AST, ALP and LDH in the serum were increased significantly (*p* <0.05) with acetaminophen administration indicating liver injury. The levels of total bilirubin also increased (*p* <0.05) by acetaminophen treatment. Co-treatment of *F. olivieri* extract (*p* <0.05) ameliorated the acetaminophen induced alterations in dose related pattern (200 and 400 mg/kg). Similarly, 50 mg/kg of silymarin erased the toxicity of acetaminophen and the values of above mentioned serum parameters reversed towards the control group. Treatment of *F. olivieri* (400 mg/kg) alone showed non significant (*p* > 0.05) variations in the above parameters.

**Table 8:** Effect of *F. olivieri* on liver marker enzymes in acetaminophen induced toxicity in rats

Treatment mg/kg bw	AST (U/l)	ALT (U/l)	ALP (U/l)	LDH (U/l)	Total bilirubin (mg/dl)
Control (Saline)	31.90 ± 2.31^d^	25.93 ± 5.11^d^	158.65 ± 3.00^d^	53.21 ± 1.46^d^	1.18 ± 0.17^c^
AMP 750	71.49 ± 2.98^a^	67.99 ± 3.01^a^	325.11 ± 2.80^a^	86.52 ± 1.58^a^	2.16 ± 0.64^a^
AMP + Sily 50	63.24 ± 1.23^b^	54.87 ± 2.67^a^	231.42 ± 2.12^b^	59.38 ± 1.34^c^	2.08 ± 0.12^a^
AMP + FOM 200	53.71 ± 2.09^b^	46.78 ± 1.76^b^	187.64 ± 2.45^c^	71.69 ± 2.03^b^	1.82 ± 0.45^b^
AMP + FOM 400	44.39 ± 2.31^c^	38.75 ± 2.45^c^	146.73 ± 1.91^d^	56.03 ± 1.32^c^	1.29 ± 0.58^c^
FOM 400	35.30 ± 1.22^d^	27.33 ± 0.91^d^	142.19 ± 2.28^d^	51.84 ± 1.11^d^	1.25 ± 0.13^c^

Mean ± SEM (06). Means with different superscript letters (^a-d^) within the column indicate significant difference (*p* <0.05). *AMP*, acetaminophen; *FOM*, *F. olivieri* methanol extract

#### Effects of *F. olivieri* on lipid profile

The cholesterol, triglyceride, HDL and LDL levels increased significantly (*p* <0.05) by acetaminophen administration to that of control rats. The administration of rats with the acetaminophen along with methanol extract of *F. olivieri* (200 and 400 mg/kg) restored these values towards the control group (Table 9). Ameliorating effects of silymarin on the lipid profile against the acetaminophen toxicity were comparable to the higher dose of *F. olivieri* (400 mg/kg). Treatment of methanol extract alone displayed non significant variation in the lipid profile as compared to the control animals.

**Table 9:** Effect of FOM on lipid profile in acetaminophen induced toxicity in rats

Treatment (mg/kg bw)	Cholesterol (mg/dl)	Triglycerides (mg/dl)	HDL (mg/dl)	LDL (mg/dl)
Control (Saline)	96.60 ± 3.12^b^	136.40 ± 2.54^c^	66.42 ± 2.54^c^	28.33 ± 1.41^d^
AMP 750	126.67 ± 3.01^a^	232.00 ± 3.02^a^	128.65 ± 1.32^a^	67.60 ± 2.68^a^
AMP + Sily 50	61.12 ± 1.31^d^	144.36 ± 1.19^c^	69.30 ± 2.07^c^	30.28 ± 0.12^c^
AMP + FOM 200	98.00 ± 3.07^b^	183.40 ± 2.27^b^	108.23 ± 1.17^b^	44.00 ± 1.15^b^
AMP + FOM 400	64.00 ± 2.12^d^	141.40 ± 2.38^c^	72.21 ± 1.56^c^	31.60 ± 1.56^c^
FOM 400	74.26 ± 1.52^c^	128.93 ± 1.15^d^	63.29 ± 1.68^d^	25.49 ± 1.85^d^

Mean ± SEM (06). Means with different superscript letters (^a-d^) within the column indicate significant difference (*p* <0.05). *AMP*, acetaminophen; *FOM*, *F. olivieri* methanol extract

#### Effect of *F. olivieri* on antioxidant enzyme armory of liver

As shown in Table 10, the acetaminophen treatment resulted in reduced activities of hepatic antioxidant enzymes; CAT, SOD, GSH-Px and GR as compared to control animals. Co-administration of *F. olivieri* extract (200 and 400 mg/kg) to acetaminophen treated rats significantly (*p* <0.05) restored the level of the above mentioned antioxidant enzymes towards the control values in liver tissue. Co-treatment of silymarin (50 mg/kg) also restored the enzyme activities in comparison to acetaminophen treated rats. Treatment of *F. olivieri* alone did not change (*p* >0.05) the activities of these enzymes.

**Table 10:** Effect of FOM on antioxidant enzyme activities in acetaminophen induced hepatotoxicity in rats

Treatment (mg/kg bw)	Catalase (U/mg protein)	SOD (U/mg protein)	GSH-Px (nM/min/mg protein)	GR (nM/min/mg protein)
Control (Saline)	5.85 ± 0.13^c^	4.23 ± 0.22^d^	114.75 ± 0.97^c^	127.53 ± 1.90^d^
AMP 750	2.44 ± 0.04^a^	1.18 ± 0.25^a^	69.82 ± 0.20^a^	75.82 ± 1.65^a^
AMP + Sily 50	5.68 ± 0.21^c^	4.32 ± 0.12^d^	116.14 ± 0.31^c^	125.31 ± 2.29^c^
AMP + FOM 200	3.95 ± 0.02^b^	2.82 ± 0.10^b^	81.52 ± 1.25^b^	97.25 ± 1.85^b^
AMP + FOM 400	5.71 ± 0.15^c^	3.78 ± 0.57^c^	110.59 ± 2.18^c^	116.84 ± 1.39^c^
FOM 400	5.89 ± 0.43^c^	4.27 ± 0.49^d^	118.72 ± 0.66^c^	129.58 ± 1.75^d^

Mean ± SEM (06). Means with different superscript letters (^a-d^) within the column indicate significant difference (*p* <0.05). *AMP*, acetaminophen; *FOM*, *F. olivieri* methanol extract

#### Effect of *F. olivieri* on biomolecules of liver

The GSH and total protein content decreased whereas the TBARS and DNA damages significantly (*p* >0.05) increased in acetaminophen treated group of rats as compared to the control rats. Co-administration of acetaminophen intoxicated rats with *F. olivieri* extract (200 and 400 mg/kg) showed the ameliorating effects on the acetaminophen induced toxicity and the level of GSH, total protein, TBARS and DNA damages restored towards the control level. Treatment of silymarin (50 mg/kg) alleviated the toxic effects of acetaminophen and restored the above parameters towards the control group (Table 11).

**Table 11:** Effect of FOM on GSH, TBARS and DNA fragmentation in acetaminophen induced hepatotoxicity in rats

Treatment mg/kg bw	GSH (μM/g tissue)	TBARS (nM/min/mg protein)	DNA fragmentation %	Total protein (μg/mg tissue)
Control (Saline)	25.52 ± 0.63^d^	1.85 ± 0.01^d^	6.12 ± 0.01^d^	4.90 ± 0.17^d^
AMP 750	10.96 ± 0.25^a^	5.17 ± 0.18^a^	32.18 ± 1.23^a^	2.58 ± 0.52^a^
AMP + Sily 50	25.01 ± 0.64^d^	1.71 ± 0.04^d^	7.11 ± 0.16^d^	3.96 ± 0.63^b^
AMP + FOM 200	17.23 ± 0.15^b^	3.63 ± 0.53^b^	22.85 ± 1.05^b^	2.87 ± 0.52^a^
AMP + FOM 400	22.76 ± 0.14^c^	2.51 ± 0.16^c^	15.42 ± 1.32^c^	3.64 ± 0.13^c^
FOM 400	25.14 ± 0.31^d^	1.21 ± 0.50^e^	8.31 ± 0.03^d^	4.45 ± 0.22^d^

Mean ± SEM (06). Means with different superscript letters (^a-e^) within the column indicate significant difference (*p* <0.05). *AMP*, acetaminophen; *FOM*, *F. olivieri* methanol extract

#### Histopathological analysis of liver

**Fig. 3:**
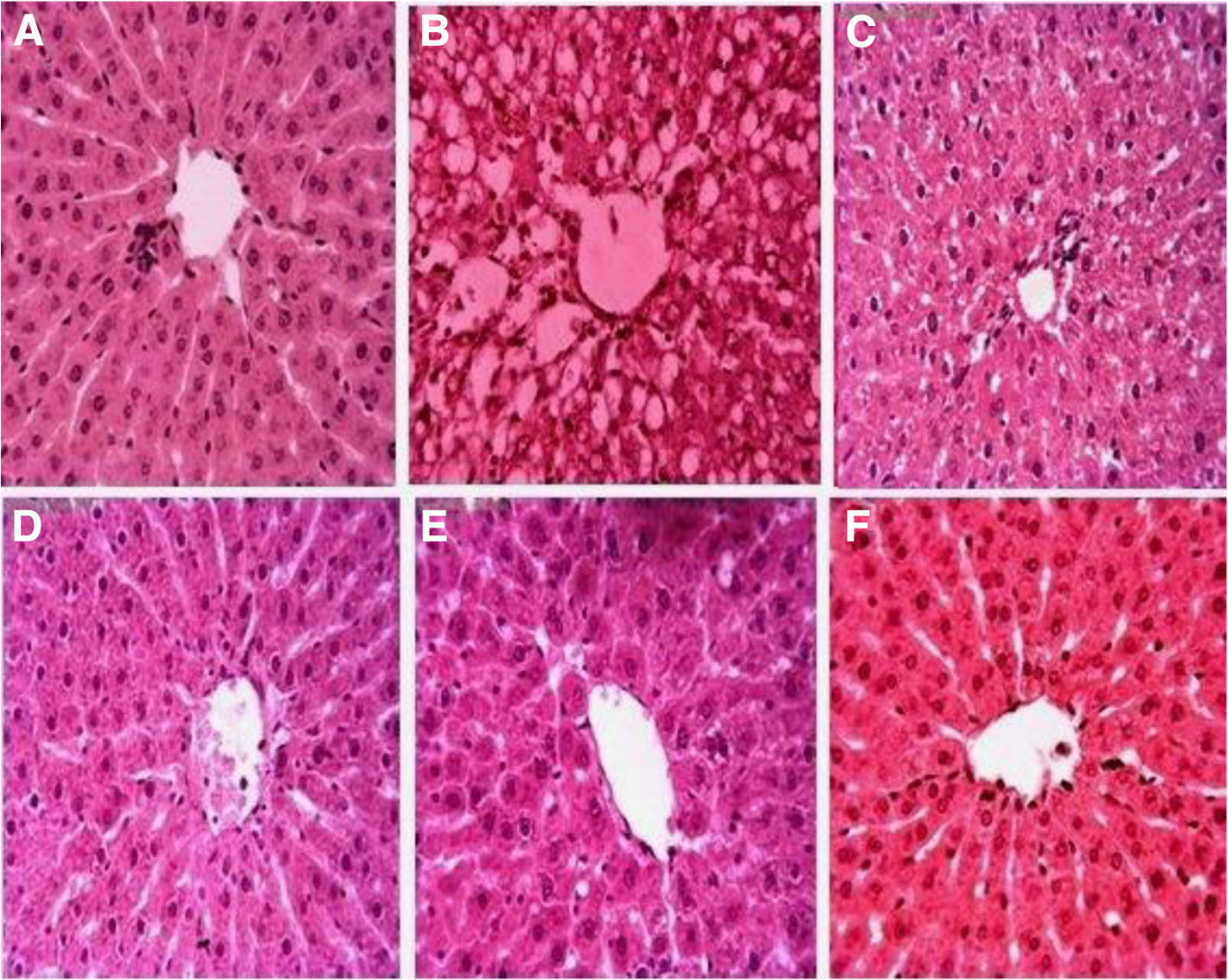
Effect of *F. olivieri* on acetaminophen induced histopathology of liver. (**a**) Control group; (**b**) Acetaminophen 750 (mg/kg) treated group, (**c**) Acetaminophen + silymarin (50 mg/kg) treated group, (**d**) Acetaminophen + FOM (200 mg/kg) treated group (**e**) Acetaminophen + FOM (400 mg/kg) treated group, (**f**) FOM (400 mg/kg) treated group

Normal hepatic architecture was observed in the liver sections of control with distinct hepatocytes surrounding the central vein with clear cell membrane and nuclear structure (Fig. 3a). Liver section of acetaminophen treated animals showed hepatic degeneration, periportal inflammation, severe centrilobular necrosis with vacuolar cytoplasmic degeneration and nuclear pycnosis around the central vein (Fig. 3b). Silymarin co-treated liver samples showed improvement in the liver architecture with mild degeneration as compared to acetaminophen treated group (Fig. 3c). Administration of *F. olivieri* extracts (200 and 400 mg/kg) results in the reduction of acetaminophen induced hepatocyte necrosis and brought the liver morphology to near normal (Fig. 3d & e). The animals treated with 400 mg/kg of the extract alone presented the normal histoarchitecture of liver (Fig. 3f).

## Discussion

The imbalance between pro-oxidants and antioxidants results into oxidative stress in the body which is promptly gaining a reputation as an important phenomenon in chronic diseases [40]. Cellular damage is the common pathway for cancer, aging and a range of diseases and antioxidants are thoroughly involved in its prevention. One of the mechanism for antioxidation is free radical scavenging action [41]. It helps the living systems to get rid of free radicals which are continuously generated and can bring about widespread damage to tissues and biomolecules [42].

In the present investigation maximum amount of total phenolic and total flavonoid contents have been recorded in the aqueous fraction of the methanol extract of *F. olivieri*. The antioxidant ability of the extract/fractions recorded in this study might be attributed by the polyphenols especially the rutin, gallic acid and catechin. Polyphenols present in plants are a group of highly hydroxylated phenolic compounds and includes hydroxycinnamate derivatives, hydroxycoumarins, flavanols, flavanones, flavonols, flavones, proanthocyanidins (tannins), anthocyanins, aurones, hydroxystilbenes, etc. The antioxidant activity of plant-derived phenolic compounds is exhibited through various mechanisms, including free radicals scavenging, metal ions chelation and anti-lipid peroxidation [43, 44].

This study has established the HPLC fingerprinting which is the best means of chemical characterization for the active phenolic acids. The polyphenol rutin, catechin and gallic acid found in the extract have been attributed with diverse pharmacological activities for instance, catechin with radical scavenging, iron chelation, cell signaling pathways and survival genes activation, regulation of ubiquitin-proteasome system and mitochondrial function [45]. Rutin exerts antioxidant, anti-inflammatory, protection against hepatotoxicity, myocardial infarction, diabetes complications, UV irradiation, and ethanol-induced gastric lesions [46, 47]. Gallic acid endorses the antioxidant and anticancer activities [48].

In this experiment the aqueous fraction exhibited strong DPPH radical scavenging ability. DPPH stable free radical method is a rapid, sensitive and easy approach to investigate the antioxidant potential of plant extracts. Hydrogen atoms exchange between the DPPH free radical and antioxidant is the basis of this assay. In this study, the highest percentage inhibition was shown by aqueous fraction as compared to others. The results indicates that the extracts may contain compounds having the ablility of donating hydrogen to a free radical for the removal of odd electron that is in charge of radical’s reactivity. Maximum scavenging potential for the DPPH radicals recorded in this investigation for the aqueous fraction might be attributed by the presence of catechin, gallic acid and other hydrogen donating antioxidants. Further, IC_50_ values obtained for DPPH scavenging assay also produced significant association (R^2^ = 0.8551; *p* <0.05, R^2^ = 0.7100; *p* <0.05) with the total flavonoid and total phenolic constituents. Our results are endorsed by a previous study where the aqueous fraction having gallic acid exhibited the strong antioxidant activity [3, 18, 49].

Superoxide anions can harm biomolecules directly or indirectly through the formation of H_2_O_2_, OH, singlet oxygen or peroxynitrite during the process of aging and pathological events like ischemic reperfusion injury; hence they are one of the most problematic of free radicals [48]. The potential of the extracts to destroy the superoxide radical produced from the PMS/NADH reaction is used to estimate the superoxide radical scavenging activity [50]. Our results revealed that all the extracts especially the aqueous fraction have shown the activity against this radical compared favourably with the standard reagent i.e. ascorbic acid indicating that the plant is an effective superoxide radical scavenger. The aqueous fraction of this plant also exhibited strong association (R^2^ = 0.8130, *p* <0.05) with the total flavonoid constituents that could be a therapeutic force of this plant against stress induced ailments. This electron scavenging potential of the aqueous and other fractions of this plant might be due its bioactive constituents and are responsible for the protection of macromolecules. Potent superoxide scavenging ability of the extract/fractions has been reported in earlier studies [3].

Total antioxidant capacity of the extracts can be assessed quantitatively by phosphomolybdenum assay. It has been established in this study that the highest antioxidant capacity for phosphomolybdate reduction is displayed by ethyl acetate fraction (IC_50_ = 78 ± 0.883 μg/ml). The results of this assay have shown the ability of the extracts to transform relative free radical species into more stable non-reactive products and to act as chain terminators [51, 52]. Phytochemical analysis of ethyl acetate fraction indicated the existence of rutin and catechin that might attribute the scavenging ability towards the antioxidant activity of *F. olivieri*.

A variety of molecules in living cells, for instance amino acids, nucleotides, lipids and sugars can react with hydroxyl radical because of its high reactivity. Thus hydroxyl radical scavenging is an essential antioxidant activity. In this study, the potential of the extracts to remove OH radicals has been linked directly with the scavenging of active species of oxygen, which reduces speed of the chain reaction. In this investigation ethyl acetate and the aqueous fraction exhibited the moderate scavenging potential for hydroxyl radicals indicating the presence of potential antioxidant phytoconstituents in *F. olivieri* [3, 18, 49].

The importance of hydrogen peroxide lies in its ability to penetrate biological membranes. Inside the cell, H_2_O_2_ can possibly react with Cu^2+^ and Fe^2+^ ions to form OH radical thus leading to several toxic effects. Some enzymes may directly inactivated by H_2_O_2_, generally by essential thiol (-SH) groups oxidation [53]. Therefore the control of H_2_O_2_ quantity that can build up is biologically beneficial for cells. In this study, methanol extract showed efficient scavenging of H_2_O_2_ that can be attributed to the presence of phenolics, which by donating electrons reduces H_2_O_2_ to H_2_O. Methanol extract and its derived fractions showed admirable antioxidant activity to eliminate the hydrogen peroxide from the system. These findings are in line with the previous studies [3, 18].

ABTS free radical-scavenging method is an excellent means to determine the antioxidant activity of a variety of substances, such as scavengers of chain breaking antioxidants or lipid peroxyl radicals and hydrogen-donating antioxidants. The extracts in our study were found to have an appreciable scavenging activity of ABTS radical; this implies that they can be useful for the treatment of pathological damage caused by radicals. The ABTS scavenging ability of the methanol extract/fractions of *F. olivieri* suggests the electron/hydrogen abilities of the constituents present in this plant. Scavenging of ABTS has also been recorded in earlier reports [18, 49].

The reductive ability of antioxidant can be measured by reducing power. The Fe^3+^ transformation to Fe^2+^ in the presence of extracts by donating an electron is the assessment of reducing power. This reducing capacity of *F. olivieri* extract/fractions which involves breakdown of free radical chain by hydrogen atom donation is an indication of potential antioxidant activities [50].

In this experiment we have evaluated the protective potential of *F. olivieri* against acetaminophen induced hepatic toxicity in rat. Liver is a vital organ to detoxify xenobiotics, chemotherapeutic agents and environmental pollutants as it is the main organ involved in metabolism and excretion. Therefore, it is subjected to a wide range of disorders and diseases. Exposure to acetaminophen causes oxidative stress and liver injury probably through the drainage of mitochondrial glutathione [10]. ROS generated during this process may result in cell death and added to the hepatic damaging actions.

Percent increase in body weight and relative liver weight of rat was significantly decreased indicating the deleterious effects of acetaminophen. This decrease in relative liver weight might reflected the hydropic degeneration, haemorrhages and necrosis linked with fatty changes. The co-treatment of FOM resulted in a rise in the relative liver weight suggesting the protective potential of phytochemicals against the damaging action of acetaminophen. The observed decrease in the blood parameters such as PCV%, Hb, MCH, MCHC, WBC and neutrophils while increase in the MCV%, TLC, PLT, %lymphocytes and %granulocytes in the blood indicated the toxic effect of acetaminophen. The decrease in the haemoglobin and its related paramters reflected the enhanced hepatic red blood cell decomposition and decreased generation of red blood cells as manifested by the enhanced level of total bilirubin in this investigation. The enhanced level of lymphocytes and granulocytes in the blood circulation with acetaminophen indicated the rise of inflammatory response. Restoration of blood parameters towards the control level with FOM indicated the therapeutic efficacy of the phyto-chemicals present in the plant.

The determination of enzymes levels for instance ALT, AST, LDH is mostly used for the evaluation of hepatic injury. Intracellular enzymes can be measured in the serum after they released into circulation due to membrane damage or necrosis. Elevated levels of AST specifies hepatic injury as alanine is converted to glutamate and pyruvate which is catalysed by ALT and released in likely manner. Hence, ALT is a better parameter to identify hepatic damage as it is more specific to the liver. The higher concentrations of enzymes in the serum indicate loss of hepatic membrane functional integrity and cell leakage. Serum ALP, total protein and total bilirubin are also linked to the liver cell function. The elevation in serum ALP is because of increased synthesis, in presence of increasing biliary pressure [19, 20, 49]. Acetaminophen administration caused a significant rise of enzyme levels including AST, ALT, ALP, total bilirubin and decline in total protein in comparison to control. *F. olivieri* methanol extract administration significantly restored these parameters in a dose dependent manner. This reversal of the enzyme levels by the extract is probably because of their membrane stabilizing activity which prevents leakage of intracellular enzymes. This is in accordance with the generally established view that serum levels of transaminases restored to normal with the regeneration of liver cells and healing of liver parenchyma [19, 50].

The failure in the normal uptake, conjugation and excretion of the total bilirubin, triglycerides and albumin by the injured liver parenchyma might cause their elevation in serum. A very high level of hypercholesterolaemia may attain in acetaminophen induced toxicity, which frequently occur in biliary obstruction [49]. The triglycerides accumulation may also due to the disturbance or inhibition of triglycerides secreting mechanism [51]. The treatment with the extract almost entirely recovered these alterations and corrected the triglycerides metabolism.

The acetaminophen induced rise in hepatic TBARS levels propose increased lipid peroxidation which leads to tissue injury and collapse of antioxidant defence mechanism to avoid excessive free radicals formation. The lipid peroxidation is frequently observed with oxidative stress. NAPQI is likely to be unable to initiate a radical hydrogen abstraction from lipid molecules. Hence, reactive oxygen species including hydroxyl radicals, superoxide anions and hydrogen peroxide are required for initiation of lipid peroxidation. Nevertheless, decrease of NAPQI, which might take place due to the presence of flavoproteins, followed by reoxidation by oxygen could give rise to superoxide anions with a subsequent formation of reactive reduced oxygen species. Even protein bound NAPQI was suggested to be prone to one electron reduction. In the toxicity mechanism of acetaminophen, lipid peroxidation is considered to be a main initiation incident [10]. *F. olivieri* treatment to the rats significantly reduced the high levels of TBARS in dose dependent manner.

One of the sensitive indices in liver cell injury is decline of superoxide dismutase enzyme activity [54]. Superoxide dismutase is one of the vital enzymes in the enzymatic antioxidant defense system. It diminishes the toxic effect caused by superoxide anion by converting it into hydrogen peroxide. *F. olivieri* causes a significant elevation in liver superoxide dismutase activity and thus lessen free radical induced oxidative injury to liver. Catalase is an antioxidant enzyme which is commonly present in all animal tissues, and the highest activity is found in liver and red blood cells. Catalase defends the tissues from highly reactive hydroxyl radicals by decomposing hydrogen peroxide. Therefore decline in the catalase activity may cause various harmful effects due to hydrogen peroxide and superoxide radical assimilation. The standard hepatoprotective drug silymarin and the higher dose of extract in our study i.e. 400 mg/kg increase the catalase activity level. Restoration of catalase activity and thereby the decrease of hepatic damage might reflect the antioxidant activity of the phytoconstituents present in the plant used in this experiment.

Glutathione (GSH), a non-enzymatic antioxidant is one of the most plentiful tripeptide found in the liver. It gets rid of the reactive oxygen species for instance superoxide radicals, hydrogen peroxide, and preserves protein thiols of membrane. In acetaminophen intoxicated rats, the reduced levels of GSH are linked with increased lipid peroxidation. *F. olivieri* administration significantly restored the level of GST and GSH-Px in a dose dependent manner. The presence of polyphenolics was disclosed in preliminary phytochemical assessment of *F. olivieri* methanol extract. The observed hepatoprotective and antioxidant activities of *F. olivieri* are may be due to the presence of polyphenols such as rutin, catechin, gallic acid and other antioxidant phyto-chemicals [55, 56].

The histopathological analysis of liver confirmed the observed modification of serum enzymatic levels to liver injury and their attributes on health. The normal hepatic tissue in our study demonstrated the usual architecture with a central vein and hepatocytes radiating from it. The portal triad consisted of hepatic artery, portal vein and bile duct which constituted various zones surrounding these areas. Acetaminophen treatment produced centrizonal necrosis, hydropic and fatty changes with sinusoids congestion. Co-treatment with *F. olivieri* repaired the liver architecture and protected the hepatic tissue from degenerative and fatty alterations, by averting the toxic chemical reaction, oxidative stress, lipid peroxidation, molecular changes in the liver tissues, micro and macro vesicular fatty changes ultimately leading to necrosis. These findings are supported by the previous hepatoprotective abilities of plant extracts [57, 58].

## Conclusion

Our study indicates that *F. olivieri* can serve as a natural source to develop the free radical scavengers which might be useful in the prevention of oxidative stress development. The significant correlation between the values of the concentration of phenolic and flavonoid compounds and antioxidant activity indicated that these compounds contribute to the antioxidant activity. Further studies should be done to isolate compounds from this plant that exhibit strong antioxidative activities. The results of in vivo study suggest that the *F. olivieri* has the ability to ameliorate acetaminophen-induced liver damage which might be associated with its antioxidant potential.

## Abbreviations

ACP: Acetaminophen
ALP: Alkaline phosphatase
ALT: Alanine transaminase
AST: Aspartate transaminase
CAT: Catalase
FOA: *Fagonia olivieri* soluble residual aqueous fraction of FOM
FOB: *Fagonia olivieri* n-butanol fraction of FOM
FOC: *Fagonia olivieri* chloroform fraction of FOM
FOE: *Fagonia olivieri* ethyl aceate fraction of FOM
FOH: *Fagonia olivieri* n-hexane fraction of FOM
FOM: *Fagonia olivieri* methanol extract
GR: Glutathione reductase
GSH: Rerduced glutathione
Hb: Hemoglobin
LDH: Lactate dehydrogenase
MCH: Mean corpuscular haemoglobin test
MCHC: Mean corpuscular haemoglobin concentration
MCV: Mean corpuscular volume
PCV: Packed cell volume
PLT: Platelet count
SOD: Superoxide dismutase
TBARS: Thiobarbituric acid reactive substances
TLC: Total leukocyte count
WBC: Whilte blood cells
